# Concomitant health benefits package design and research prioritisation: development of a new approach and an application to Malawi

**DOI:** 10.1136/bmjgh-2021-007047

**Published:** 2021-12-11

**Authors:** Laetitia Schmitt, Jessica Ochalek, Karl Claxton, Paul Revill, Dominic Nkhoma, Beth Woods

**Affiliations:** 1 Centre for Health Economics, University of York, York, UK; 2 Health Economics and Policy Unit, Kamuzu University of Health Sciences, Lilongwe, Malawi

**Keywords:** health economics, health services research, public Health

## Abstract

Health benefits packages (HBPs) are increasingly used in many countries to guide spending priorities on the path towards universal health coverage. Their design is, however, informed by an uncertain evidence base but research funds available to address this are limited. This gives rise to the question of which piece of research relating to the cost-effectiveness of interventions would most contribute to improving resource allocation. We propose to incorporate research prioritisation as an integral part of HBP design. We have, therefore, developed a framework and a freely available companion stand-alone tool, to quantify in terms of net disability-adjusted life-years (DALYs) averted, the value of research for the interventions considered for inclusion in a package. Using the tool, the framework can be implemented using sensitivity analysis results typically reported in cost-effectiveness studies. To illustrate the framework, we applied the tool to the evidence base that informed the Malawi Health Sector Strategic Plan 2017–2022. Out of 21 interventions considered, 8 investment decisions were found to be uncertain and three showed strong potential for research to generate large health gains: ‘male circumcision’, ‘community-management of acute malnutrition in children’ and ‘isoniazid preventive therapy in HIV +individuals’, with a potential to avert up to 65 762, 36 438 and 20 132 net DALYs, respectively. Our work can help set research priorities in resource-constrained settings so that research funds are invested where they have the largest potential to impact on the population health generated via HBPs.

**Summary box**

- Evidence on the costs and benefits of healthcare interventions considered for inclusion in a health benefits package (HBP) is inevitably uncertain.
- Methods such as value of information analysis can be used to guide research priorities, but their application requires detailed knowledge of statistical theory and the availability of data that are often not reported in the published cost-effectiveness studies that form the evidence base for HBP design. As a result, there is currently no framework in place to help align research strategies with the evidential needs of HBPs.
- We provide a framework with an easy-to-use accompanying Value of Information for Health Benefits Package design (VOI-HBP) tool to estimate the health benefits of undertaking research to inform the inclusion and exclusion of interventions within HBPs. These can be used to prioritise the allocation of research funds. The tool only requires data that are generally available from published cost-effectiveness studies.
- Application of the framework and VOI-HBP tool to the evidence base that informed the Malawi Health Sector Strategic Plan for 2017–2022 identified three interventions to consider when establishing healthcare research priorities: ‘male circumcision’, ‘community-management of acute malnutrition in children’ and ‘isoniazid preventive therapy in HIV +individuals’.
- Our work provides a practical approach that can be used alongside HBP design to guide the allocation of research funds to where these can have the largest benefits in improving population health.

## Introduction

To achieve Universal Health Coverage of their populations^1^ using a limited resource envelope, governments of low- and middle-income countries (LMIC) inevitably must make hard choices when selecting the healthcare interventions that their population has access to, free-of-charge, through publicly pooled funds.

Recent advances in methods to identify the set of interventions to be provided, that constitute what is known as a Health Benefits Package (HBP), recommend explicit consideration of the population net health effect (pNHE) associated with including in the package each intervention under consideration.^2 3^ This metric accounts for both the health expected to be generated by the intervention, and the losses in health associated with funding the intervention rather than addressing other healthcare priorities. The assessment of the pNHE of relevant interventions is informed by data coming from a range of sources. National surveys and bottleneck analyses are often used to assess population needs and interventions’ capacity to cover them, whereas estimates of per patient cost and health benefit typically come from published cost-effectiveness analyses.

Cost and health benefit estimates are, however, uncertain, due to limited data availability on key quantities such as drug and programme costs, clinical effectiveness and its long-term implications for patients’ quality of life and life expectancy.^3 4^ Approaches to quantifying uncertainty in the cost-effectiveness of interventions considered for inclusion in HBPs have been proposed and used, such as stochastic league tables whereby interventions are ranked by their probability of being cost-effective.^5^ These approaches, however, fall short of guiding policy makers as to how they should respond to uncertainty, that is whether this uncertainty matters to the resource allocation process and should be addressed by funding further research. As a result, research strategies may not be aligned with the evidential needs of HBPs. We, therefore, propose a framework to support the concomitant design of HBPs and research prioritisation, based on an assessment of where additional evidence would be most valuable in generating health gains.

Our framework builds on value of information (VOI) methods^6^ to quantify the pNHE of reducing uncertainty through further research. The contribution of our work is to tailor the application of VOI methods to the evidence base that typically informs HBPs, namely secondary cost-effectiveness data.

VOI methods or other methods similarly grounded in statistical decision theory, are often used to determine how much one may lose out by taking a decision with uncertain outcomes and therefore how much benefit there is to reducing uncertainty via research.^6 7^ Their application usually requires estimates of uncertainty around outcomes, or around the quantities that drive them, to produce probabilistic simulations of the values that outcomes could take.^8 9^ This data is, however, not readily available from published cost-effectiveness studies that overwhelmingly constitute the evidence base informing HBPs. The high burden of information inputs and technical knowledge associated with the application of statistical decision theory methods have impeded the use of VOI methods for aligning research funds allocation with evidential requirements.

We have, therefore, developed a freely available tool—the Value of Information for Health Benefits Package design (VOI-HBP) tool—that addresses these two hurdles and estimates the value of research using only the information that is typically reported in cost-effectiveness studies and without the need for undertaking supplementary statistical analysis. Our framework and companion VOI-HBP tool aim to provide a practical approach to informing deliberations about research priorities in LMIC countries so that the limited resources allocated to HBPs achieve greater gains in population health.

We apply the VOI-HBP tool to the evidence base that informed the HBP of Malawi’s Health Sector Strategic Plan (HSSP) 2017–2022^10^. Malawi has used a HBP since the early 2000s to prioritise interventions for funding within its national health budget. In addition, clear ambitions have been set to secure funds for health research, with the government agreeing to commit 2% of its overall health budget to research activities and overseas development partners being required to commit 5% of their assistance funds.^11 12^ Malawi’s National Health Research agenda^12^ is developed through structured deliberations of a large panel of experts and stakeholders from the Ministry of Health, academic institutions, service providers and bilateral and multilateral donors. Alongside several criteria such as equity impacts and public acceptability of research, these deliberations are informed by qualitative assessments of evidence needs, where particular attention is given to evidence gaps on efficacy. Whether these identified evidence gaps translate to uncertainty in cost-effectiveness and could challenge the current allocation of limited resources is, however, not considered. In this context, the Ministry of Health^13^ acknowledges the need to strengthen these deliberations with quantitative estimates of where research could make the largest contributions to population health.

## A framework to prioritise research: quantifying the health benefits from better evidence

### Core principles

The central tenet of the framework is that, by resolving uncertainty around which healthcare interventions would be expected to improve net population health if invested in, and which would not, research can improve HBP design and thereby generate additional population health gains. This section describes the principles underpinning the quantification of these gains (or losses), summarised as pNHE and shows how this can guide research prioritisation.

The decision to include an intervention in a HBP can be informed by an assessment of its expected pNHE, which encompasses the total health generated, net of the health opportunity costs imposed by funding it, and is often expressed in net disability-adjusted life-years (DALYs) averted.^3^ Interventions that are expected to generate a positive pNHE, that is to provide more health than would have been generated if the resources invested had instead been used to fund other interventions, would thus be potentially included in the package. These interventions are described as being cost-effective. Conversely, interventions that are expected to generate a negative pNHE, since their costs impose too high health opportunity costs compared with the health gains they offer, are not cost-effective.

**Figure 1:**
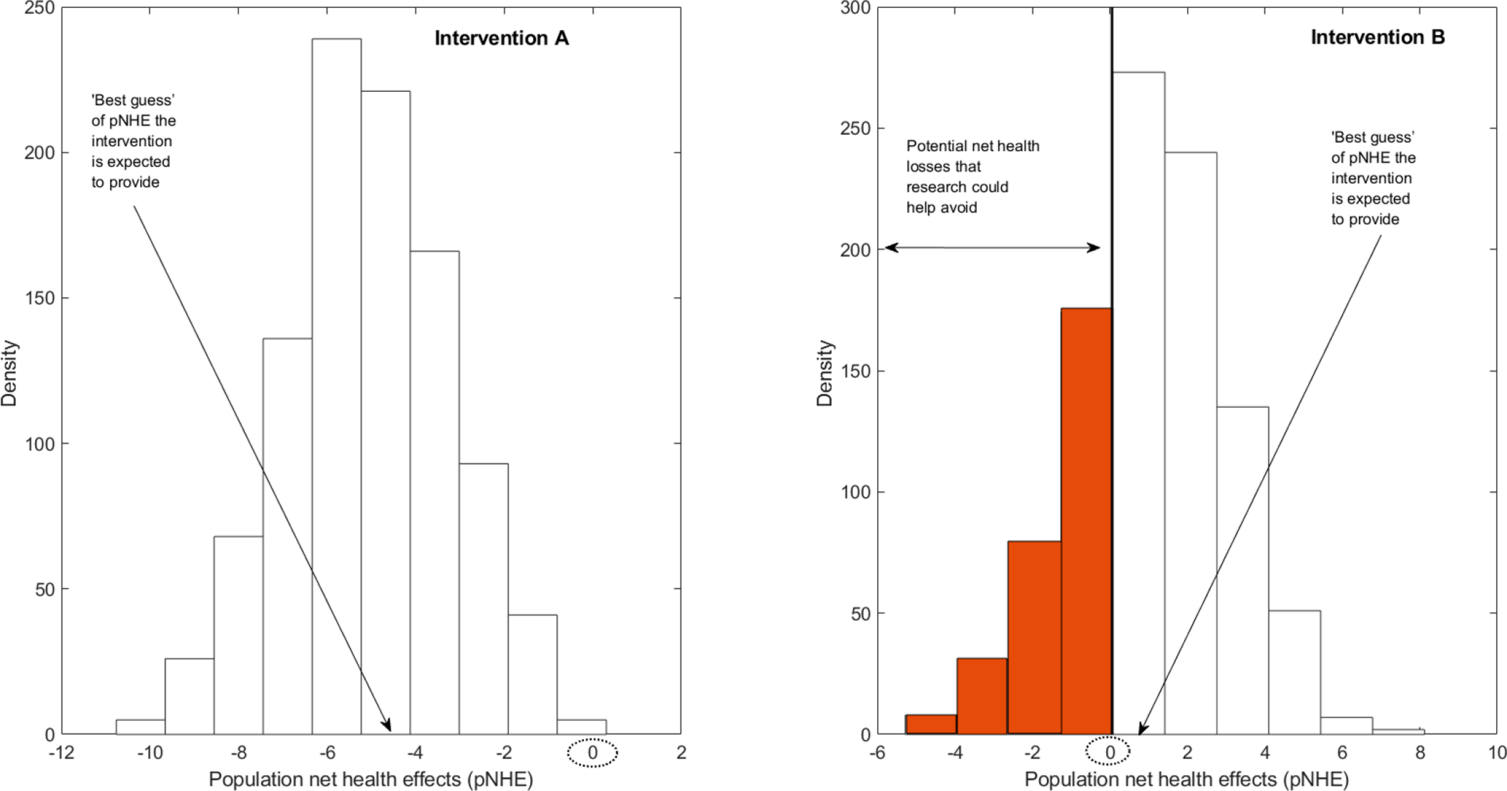
Assessing the value of research in population net health effects (pNHE).

However, there is typically uncertainty about the magnitude of pNHE expected to be achieved by each intervention as the set of clinical, epidemiological and economic quantities that underpin this calculation (eg, treatment effect size, baseline survival rate and quality of life, prevalence of disease, cost of care) are uncertain themselves. This level of uncertainty can be described by a probability distribution that shows the likelihood that the pNHE estimate could take a range of values, as is illustrated in figure 1. Linking together each possible value the pNHE estimate could take and the likelihood of each value, we can derive a ‘best guess’ of how much population net health an intervention would be expected to generate. In figure 1, the ‘best guess’ of pNHE is negative for intervention A (ie, intervention A is not expected to be cost-effective) and positive for intervention B (ie, intervention B is expected to be cost-effective). For both interventions we are unsure whether the ‘best guess’, formulated based on currently available evidence, will be the true outcome. For intervention A, however, a net health loss is expected even in the current most optimistic scenario. In other words, despite substantial uncertainty around future outcomes, it is fairly certain intervention A will not be cost-effective. On the contrary, for intervention B, the range of plausible pNHE values spans from net health losses to net health gains, indicating uncertainty in whether, on the basis of pNHEs, the intervention should be excluded or included from the package.

**Figure 2:**
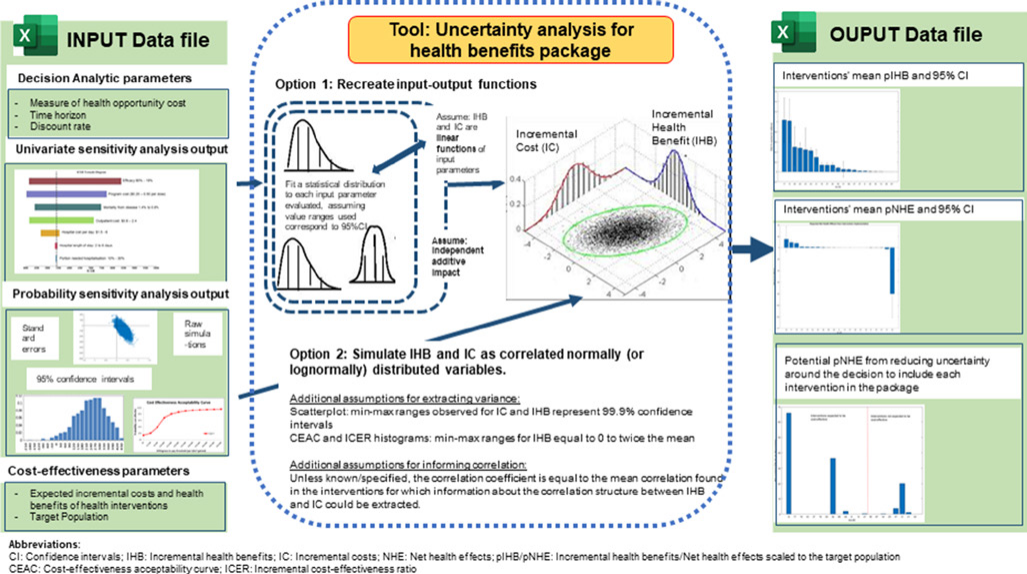
Tool's input data requirements and statistical methods and assumptions underpinning the computation of uncertainty ranges around pIHB and pNHE and of the value of research.

If currently available evidence can support two courses of action, there is a risk of taking the wrong decision. A second step is therefore required to assess whether this risk is tolerable or should be reduced. Such an assessment can be undertaken by applying VOI methods^6^ and involves the quantification of the consequences of taking the wrong decision. For intervention B, which is currently expected to be cost-effective, these consequences correspond to the net health losses incurred by incorrectly including this intervention in the package; they are represented by the shaded part of the pNHE distribution.

By combining an assessment of the potential negative effects on population net health of making an incorrect investment decision with an assessment of their likelihood, it is possible to estimate how much population net health research could generate. If, for instance, additional evidence was to suggest that implementing intervention B would most likely generate a net health loss, it would not be included, and a net health loss would thereby be avoided. Conversely, if an intervention was originally expected to be cost-ineffective but was subject to further research and found to offer net health gains, the research and subsequent updating of the HBP could allow access to a cost-effective intervention.

The greater the magnitude and likelihood of the potential net health losses associated with taking a decision informed by imperfect evidence, the more pNHE research can generate. The pNHE of research is indeed expected to be much larger for intervention B, for which potential net health losses from incorrectly including it in the HBP are substantial and quite likely, than for intervention A, for which the likelihood of forgoing any net health gain by excluding it is near zero. By undertaking this assessment for each of the interventions considered for inclusion in the package, it is possible to identify which interventions research would be most valuable for.

### Generic data requirements

The pNHE metric can be derived from population-level estimates of the incremental costs and incremental health benefits of implementing the intervention compared with the status-quo, combined with three quantities: (1) the period over which interventions are expected to be delivered once included in a package; (2) the discount rate used to adjust the values of costs and benefits occurring in the future and (3) the health opportunity cost of spending limited healthcare funds that represents the rate at which incremental costs are converted into health forgone elsewhere in the healthcare system. To quantify the uncertainty around the pNHE to be generated by each intervention, these three decision-analytic parameters need to be linked with probability distributions (as opposed to point estimates) of the incremental costs and incremental health benefits scaled to the population groups that stand to receive each intervention.

## The VOI-HBP tool to support application of the framework

Since probability distributions of incremental costs and incremental health benefits are usually not available from published cost-effectiveness studies, the uncertainty around each intervention’s expected pNHE and an estimate of the health benefits from improved evidence cannot be easily quantified. We, therefore, developed the VOI-HBP tool to bridge this data gap and perform VOI analysis to quantify the pNHE that a better-evidenced HBP could generate. The tool exploits sensitivity analysis results reported in the cost-effectiveness literature to produce probability distributions of incremental costs and incremental health benefits, based on which it derives a probability distribution of pNHE and, using VOI analysis, generates estimates of the pNHE from research expressed in DALYs. The VOI-HBP tool and a guide for use are available at: https://www.york.ac.uk/che/research/global-health/methods-guidelines/%23tab-5.

A summary of the VOI-HBP tool’s input data requirements and output is provided in figure 2. Three sets of input are to be collated into a single Excel worksheet to be uploaded to the VOI-HBP tool. The first set consists of the three decision-analytic parameters listed in the generic data requirements section that are common to all interventions (analytic time-horizon, discount rate(s) and health opportunity cost estimate). The second set consists of ‘best guesses’ point estimates of incremental health benefits, incremental costs and target population for each intervention. The third set consists of sensitivity analysis results reported in cost-effectiveness studies. To ensure wide usability, the tool only requires input that can be relatively easily read off from graphs, plots or tables (see user-guide for more details).

Sensitivity analysis results of cost-effectiveness studies may be reported using a wide range of metrics and media beyond conventional confidence intervals or standard errors. For instance, univariate sensitivity analysis results, where changes in incremental cost and benefit are observed in response to changes in selected key input quantities evaluated one at a time, are typically reported by tornado plots. In contrast, probabilistic sensitivity analysis results, where probability distributions are fitted to each input parameters that are varied simultaneously and randomly sampled, can be reported via: (1) scatterplots of the resulting simulations of incremental costs against incremental benefits; (2) histograms of incremental cost effectiveness ratios or (3) cost-effectiveness probabilities for different willingness-to-pay values for a DALY averted (or other summary metric of health outcomes). Using a range of assumptions and statistical methods tailored to the type of metrics and medium used for reporting sensitivity analysis results, the VOI-HBP tool extracts for each intervention information on estimates of correlation and variance of incremental costs and incremental health benefits to fit probability distributions and perform a probabilistic simulation of costs and benefits. It then expresses simulated costs as health losses based on the health opportunity cost estimate of spending limited healthcare funds and combines them with simulated health benefits to generate a probability distribution of net health effect. The latter is then scaled to the target population that is to benefit from the intervention, over the time-horizon that it is expected to remain in place.

For those interventions without any sensitivity analysis data, the tool provides an exploratory analysis of the potential value of research. It simulates a probability distribution of pNHE using variances and correlation estimates that are equal to the average of the values extracted for the set of interventions for which a sensitivity analysis output was available. A summary of the VOI-HBP tool’s statistical methods and assumptions is described in figure 2 and the complete description is provided in the user-guide.

## An application to Malawi’s HBP

### Data inputs

For 67 interventions considered in the HBP of Malawi’s HSSP (2017–2022), ‘best guess’ point estimates of (1) incremental costs and incremental health benefits (in DALYs) against a ‘do-nothing’ alternative and (2) population subgroups that stand to benefit from interventions (taking into account barriers to access and care demand) were available.^3^ Costs and benefits point estimates came from studies of the Tuft Global Health Cost-Effectiveness registry and WHO’s CHOosing Interventions that are Cost-effective dataset whereas target population sizes for each intervention and their realistic levels of coverage came from the Clinton Health Access Initiative.

Of those 67 interventions, the costs and benefits of 21 interventions were informed by cost-effectiveness studies that reported sensitivity analyses results. We applied our framework to this subset of 21 interventions. The type of sensitivity analysis results reported is described in table 1 and the nature of the information extracted and collated into the tool’s input data file is described in the user-guide.

**Table 1:** Type of analyses undertaken and medium used to report results in the subset of 21 interventions for which a sensitivity analysis around cost-effectiveness results had been undertaken

Type of sensitivity analysis	Medium used to report results	Numbers of interventions
Univariate	Table of incremental costs and benefits under single parameter changes	5
	Tornado plot of ICERs under single parameter changes	2
Probabilistic	Raw simulations	2
	Scatterplot of incremental costs vs incremental benefits	1
	Histogram of ICER	1
	Cumulative distribution of ICER	1
	Confidence intervals around mean incremental costs and benefits	8
	SEs around mean incremental costs and benefits	1

ICER, incremental cost effectiveness ratio

Interventions were assumed to remain in place for 20 years and costs and health benefits were discounted at 3% per annum.^14^ In line with previous work on Malawi HBP,^3^ we used a measure of health opportunity cost of US$61/averted DALY that is, for every US$61 spent on an intervention, one averted DALY would be displaced elsewhere in the healthcare system.

### What drives uncertainty in whether to include an intervention in the HBP?

**Figure 3:**
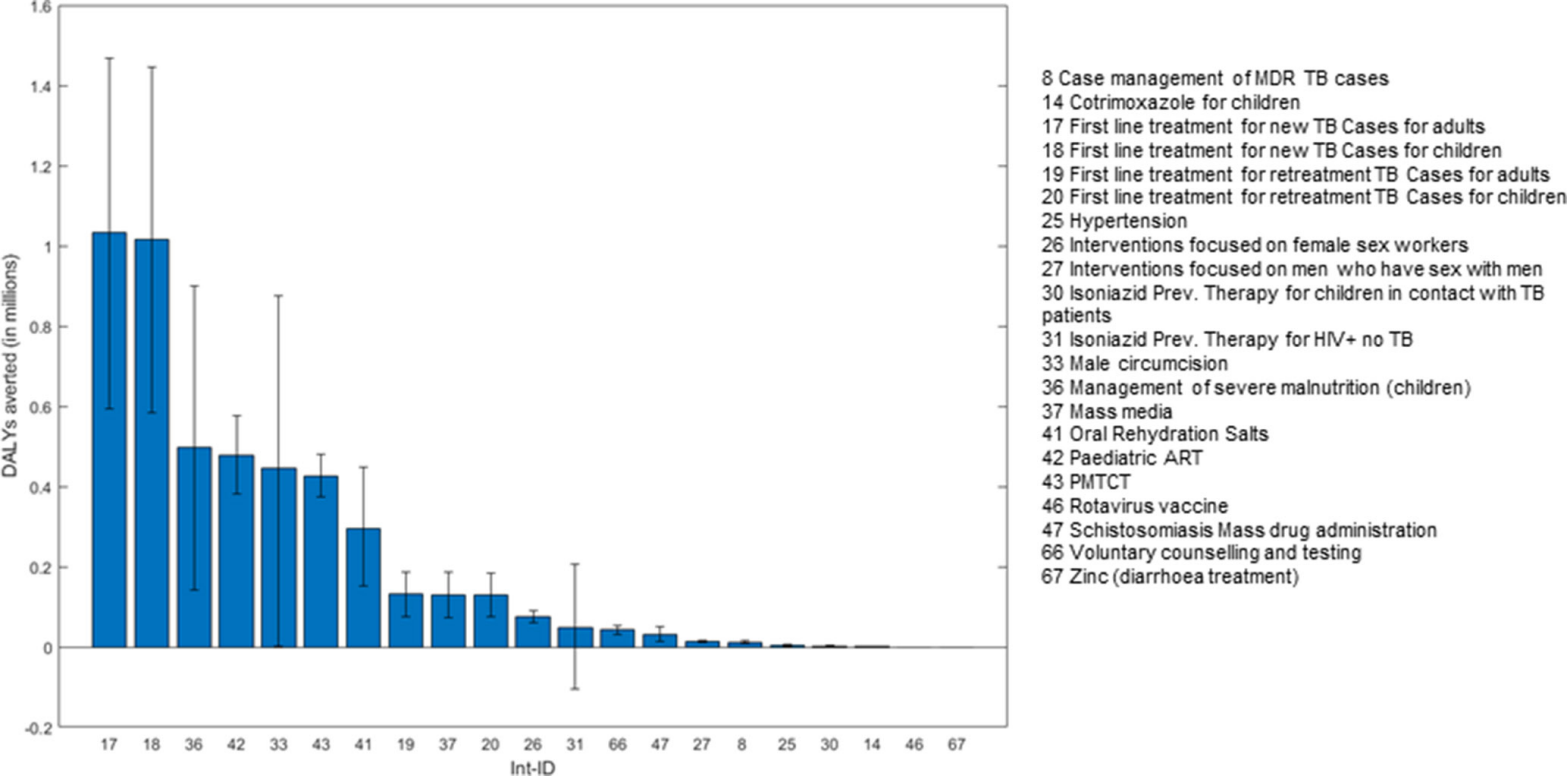
Uncertainty around the expected population incremental health benefits from implementation. DALY, disability-adjusted life-years; MDR, Multidrug resistant; TB, Tuberculosis; ART, Antiretroviral therapy; PMTCT, Prevention of mother-to-child transmission.

**Figure 4:**
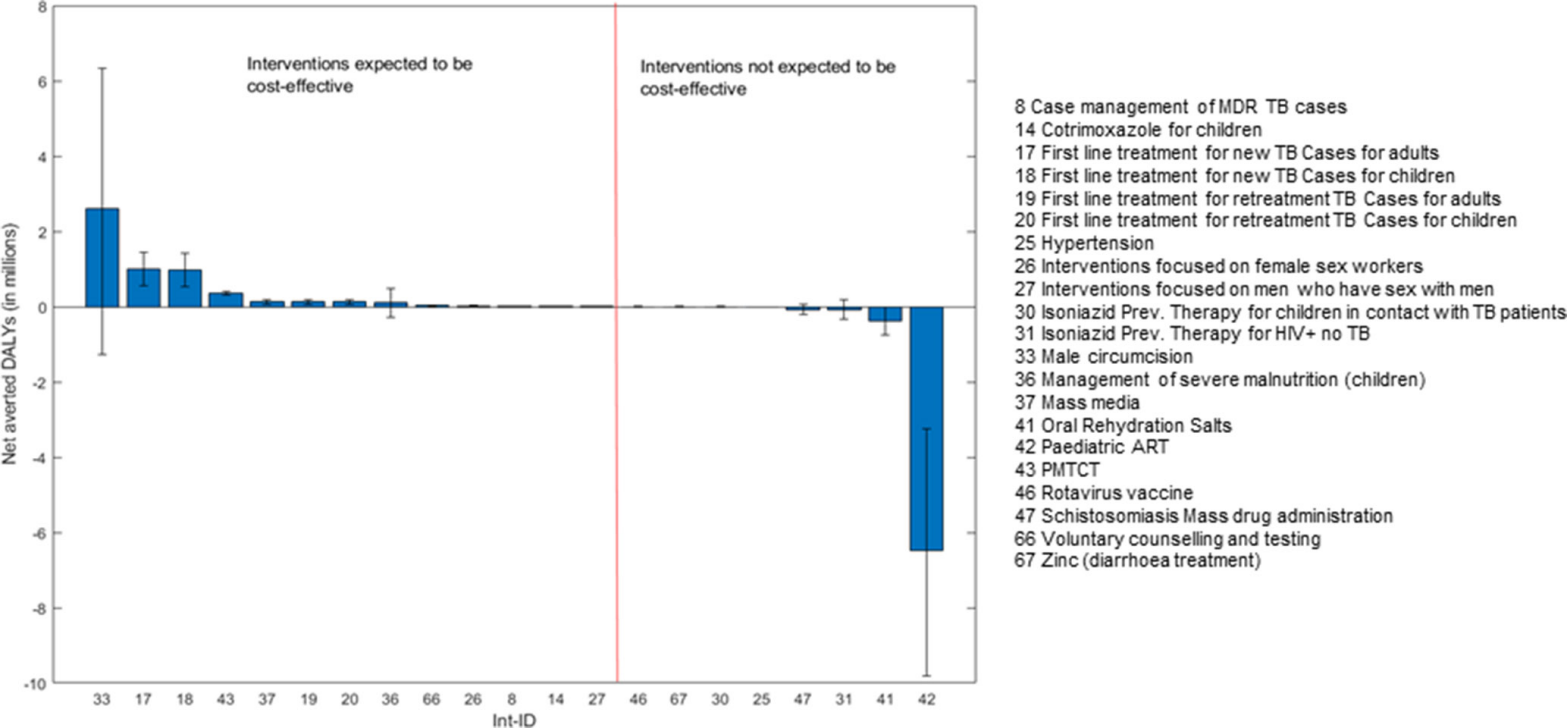
Uncertainty around the expected population net health effects from implementation. DALYs, disability-adjusted life-years; MDR, Multidrug resistant; TB, Tuberculosis; ART, Antiretroviral therapy; PMTCT, Prevention of mother-to-child transmission.

Figures 3 and 4 rank the 21 interventions by expected population incremental health benefit and pNHE respectively, discounted over the 20-year time horizon, where error bars indicate uncertainty. Comparison of the two rankings underlines the importance of informing investment decisions by considering the health opportunity costs imposed by funding interventions alongside expected incremental health benefits, as opposed to solely focusing on the latter. For instance, while figure 3 shows that the population incremental health benefit of ‘paediatric antiretroviral therapy (ART)’ (intervention (int.) 42) is expected to be sizeable, figure 4 indicates that these benefits would not offset the health displaced elsewhere in the Malawi healthcare system by funding this intervention which, overall, would imply a net burden of 6.5 million additional DALYs. Overall, among the 21 interventions considered, 13 are expected to generate a positive pNHE, that is, to be cost-effective, with the top ‘best buys’ including ‘male circumcision’ (int. 33 with 2.6 million net DALYs averted), and ‘first-line management of new cases of tuberculosis (TB)’ in adults and in children (int. 17 and int. 18 expected to avert a combined 2 million net DALYs).

Comparison of figures 3 and 4 also indicates that uncertainty around effectiveness may be a poor indicator of uncertainty around the investment decision and thus, of the need for further research. For instance, while errors bars indicate substantial uncertainty around the magnitude of incremental health benefits from providing first-line treatment to new tuberculosis cases (int. 17 and int. 18), lower-bound estimates of pNHE suggest that, even in a pessimistic scenario, this would avert a minimum of 1.1 million net DALYs across adults and children. There is therefore little uncertainty around the decision to include these interventions in the package.

A wide range of pNHE does not, however, necessarily equate with high decision uncertainty. Decision uncertainty arises if the range of pNHE spans across net health losses to net health gains and thereby could support both inclusion and exclusion of the intervention in the package. This is the case for eight interventions (int. 33, 36, 26, 27, 30, 47, 31, 41) where the error bars around expected pNHE cross the x-axis of no effect in figure 4. This is particularly apparent for three interventions: ‘male circumcision’ (int. 33), ‘community-management of acute malnutrition in children (CMAM)’ (int 36), and ‘isoniazid preventive therapy in HIV +individuals no TB’ (int. 31). While the first two are expected to be cost-effective (expected pNHE >0), the latter is expected to be cost-ineffective (expected pNHE <0).

### Where is research to support HBP design most valuable?

**Figure 5:**
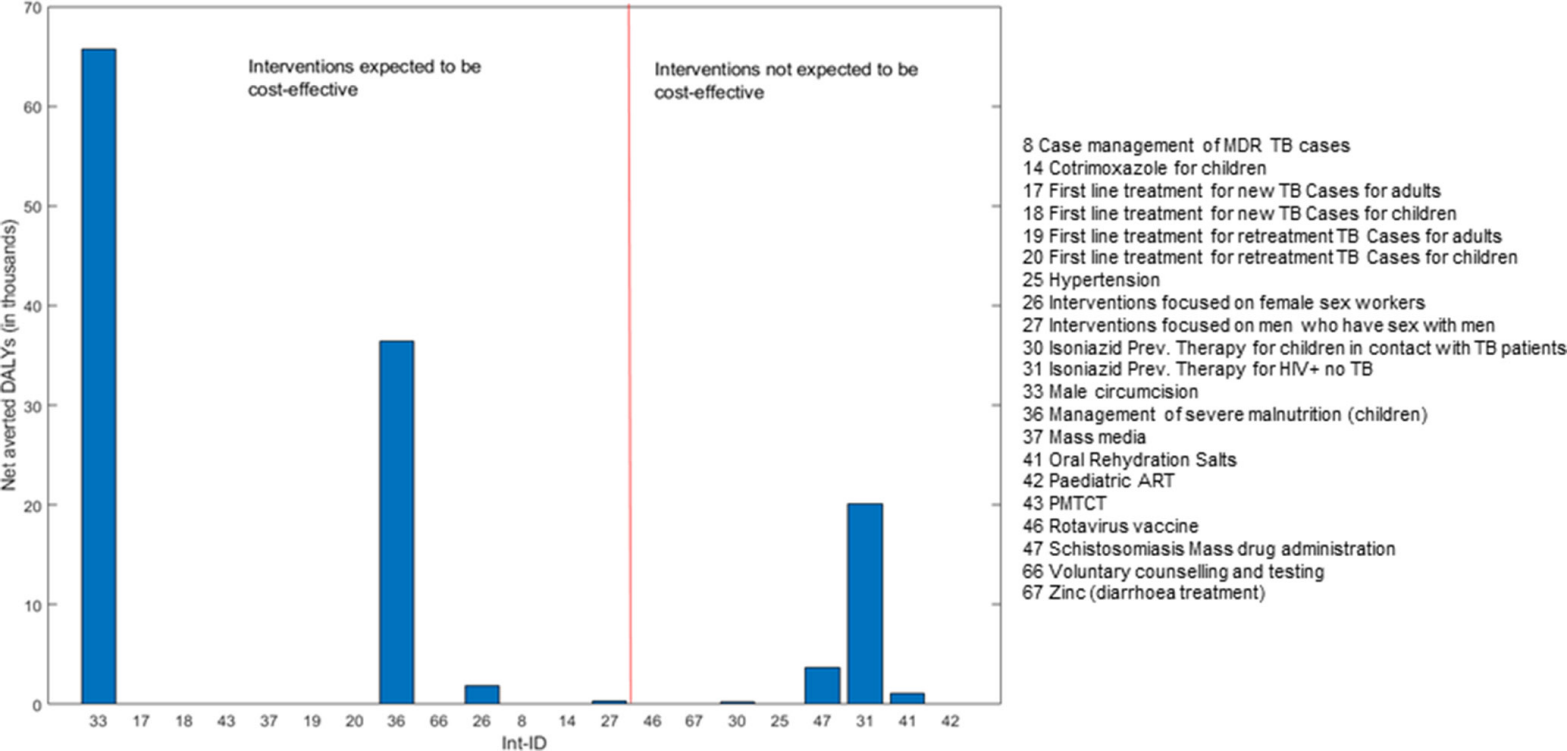
Potential population net health effects from research. DALYs, disability-adjusted life-years; MDR, Multidrug resistant; TB, Tuberculosis; ART, Antiretroviral therapy; PMTCT, Prevention of mother-to-child transmission.

Once the interventions for which there is uncertainty around the investment decision have been identified, the crucial step is to evaluate whether this uncertainty is consequential to HBP design and thus, worth reducing. Figure 5 depicts the maximum net health gains that could be generated by research if it were to resolve the uncertainties around whether to include each of the 21 interventions considered for the package. These estimates of the value of research follow interventions’ ordering by expected pNHE to visually help identify: (1) the expected ‘best buys’ for which there is substantial value in reducing uncertainty around whether these interventions should be included within the HBP and (2) interventions for which there is potential value in further researching whether excluding them from the HBP is, indeed, the appropriate decision.

Comparison of figures 4 and 5 shows that, although ‘male circumcision’ is expected to be a ‘best buy’, implementing it could lead to potentially large net health losses. As a result, resolving uncertainty around the decision to invest in this intervention could potentially avert up to 67 762 net DALYs. In contrast, there is unsurprisingly no value in undertaking research on ‘first line management of new cases of tuberculosis’ in either adults or children since, as shown in figure 4, expected pNHE values for interventions 17 and 18 only span across net health gains.

Among the other interventions for which the presence of decision uncertainty had been identified, figure 5 shows that resolving uncertainty around the decision to invest in CMAM (int 36)—currently expected to be cost-effective—and the decision to exclude ‘isoniazid preventive therapy for HIV +individuals no TB’ (int 31)—currently expected to be cost-ineffective—could help avert up to 36 438 and 20 132 net DALYs, respectively. In summary, by applying the framework to the evidence presented in existing cost-effectiveness studies, we have identified that out of 21 interventions considered for the package, eight decisions about whether to an include an intervention within the HBP were uncertain and three (int 33, 36 and 31) showed potential for research to generate large health gains.

Exploratory analysis results including the 46 interventions for which sensitivity analysis data was not available is provided in the online supplemental appendices. This indicates that several interventions for which no sensitivity analysis results were presented may well represent priority areas for further research. In particular, research on the cost-effectiveness of two maternal interventions: management of obstructed labour (int 34) and caesarean section (int.10) could deliver substantial health gains (above 200 000 DALYs, respectively). This finding is, however, conditional on the assumption that the uncertainty around the incremental health benefits and incremental costs of these two interventions is of similar magnitude to the one found on average among the subset of 21 interventions for which sensitivity analysis data was available. This present exploratory analysis should therefore be complemented with expert opinions and/or a deeper search of the evidence base but it usefully outlines those interventions such efforts may need to focus on.

10.1136/bmjgh-2021-007047.supp1 Supplementary data

### What are the implications of a changing evidence base and uncertain measures of health opportunity cost?

Costs of treatment and baseline life expectancy are key drivers of cost-effectiveness that deserve particular attention as they are subject to change over time and may therefore require refining. For instance, the cost-effectiveness study^15^ that informs ‘paediatric ART’ (int. 42) assumes a yearly per-patient treatment cost of $1436 whereas recently reported prices of ART treatment in Malawi were around $100 per person.^16^ By combining information from both studies, the lifetime per-patient incremental cost of providing paediatric ART would drop from US$11 660 to US$1115. While our best guess of pNHE would remain negative, the decision to exclude paediatric ART from the package would no longer be so clear-cut as expectations of pNHE would now range from −544 761 to 140 400 net DALYs averted. In this context, undertaking research could help avert up to 13 000 net DALYs. In addition, in several studies of paediatric interventions, incremental health benefits were computed assuming a life expectancy at birth that is at least 10 years lower than the current estimate of 66.8 years for Malawi. For these interventions where costs are incurred in early years, but benefits can be reaped throughout a lifetime, a considerable lengthening of life expectancy will impact the magnitude of incremental health benefits, the extent of which will, however, depend on the discount rate applied.

The range of pNHE expected to be generated by each intervention intrinsically depends on the measure of health opportunity cost. This can be interpreted as how much a country can afford for averting a DALY and should ideally reflect the marginal productivity of the healthcare system^17^ but the latter is expected to be uncertain. Since the larger the opportunity cost estimate, the more the HBP would expand, its validity could be pragmatically gauged based on the proportion of a country’s healthcare budget that would be devolved to the HBP. While our current estimate of US$61/averted DALY may seem low,^18^ increasing it did not seem appropriate since at that value, the total HBP cost was already greater than the resources budgeted for it.^3^

## Conclusions

We propose a framework to concurrently support HBP design and research prioritisation by quantifying the improvement in population net health benefits to be generated by a better-evidenced HBP. To overcome the technical and data-related barriers to the implementation of our framework, based on VOI methods, we have developed a stand-alone easy-to-use quantitative tool that provides estimates of the maximum potential population health benefits of research using only sensitivity analysis results reported in cost-effectiveness studies. Using this tool, application of the framework to Malawi’s HSSP helped identify three interventions for which there was a high degree of uncertainty about whether including them in the HBP would generate positive or negative pNHEs. Across these interventions—male circumcision, CMAM and isoniazid prevention therapy in people living with HIV—research offers the potential to avert an estimated 122 000 DALYs.

By facilitating the production of quantitative estimates of the health benefits of research, we hope this work can provide an important input into prioritising the allocation of research funds. This in turn can help to ensure that restricted healthcare resources are put to their best use and achieve greater health gains in populations of LMICs. This type of quantitative information also provides a basis for communication between countries and international research funders about interventions where further research is potentially valuable. In previous work we have shown how specific research proposals that address key uncertainties relating to the costs and effects of these interventions can be evaluated.^19^

Our VOI-HBP tool is hoped to enable a wide application of the framework so that analysts within and supporting health ministries can: (1) identify the interventions which should be prioritised for funding based on current available evidence and (2) establish research priorities to address evidential requirements pertaining to cost-effectiveness, using simply secondary cost-effectiveness data at hand and without undertaking any additional statistical analysis. While the tool circumvents the high data and statistical burden that would normally be required to implement the framework, its computations inevitably rely on a set of structural assumptions regarding statistical distributions and underpinning parameters, in particular when data on correlation and minimum and maximum values of incremental benefits are not available. These assumptions can, however, be easily modified or removed to test for results’ sensitivity to them. In the present application of the framework to the evidence base used for designing Malawi’s HBP, additional analysis provided in online supplemental appendices suggests the list of interventions with the largest potential for generating health gains from research was not sensitive to the assumptions used.

Analytical results generated by the application of the framework are, however, intended to support rather than prescribe decisions. They should be carefully appraised in light of: (1) the quality and relevance of the cost-effectiveness evidence base, including whether key drivers of cost-effectiveness findings need refining or updating and (2) results’ sensitivity to the choice of decision-analytic parameters such as the estimate of the health opportunity cost of spending healthcare funds.

In addition, the framework proposed focuses on health maximisation whereas stakeholders may consider wider social objectives when appraising the value of research studies. In the development of Malawi’s HSSP 2017–2222, an extensive consultation underlined the need to consider health effects alongside four additional objectives: reducing health inequalities and promoting financial protection, exceptional donor funded interventions, continuation of care and complementarities between interventions. Nevertheless, by quantifying how much population health research could generate by improving HBP design, our framework can provide a valuable input to deliberative research prioritisation processes that consider a wider range of social objectives. In Malawi, a National Health Research Taskforce, coordinated by the Department of Planning of the Ministry of Health, has been established to define research priorities to inform delivery of its next HSSP 2022–2027, to which the revised HBP will be central.^13^ The Taskforce is to play a key role in guiding the allocation of research funds coming from the government and from several overseas donor-channels. It is anticipated that our framework will provide valuable quantitative insight to help shape the new HSSP and support the Taskforce in setting out research priorities.
