# Supplementary material for: Concomitant health benefits package design and research prioritisation: development of a new approach and an application to Malawi
Source: BMJ Glob Health. 2021 Dec 11;6(12):e007047. doi: 10.1136/bmjgh-2021-007047 (PMC8671930; doi:10.1136/bmjgh-2021-007047)
Supplement: Supplementary data [file bmjgh-2021-007047supp001.pdf]

Supplementary Appendices

Abbreviations:

IHB: Incremental health benefits;

IC: Incremental cost; pNHE:

corr: correlation coefficient between IHB and IC;

pNHE: population Net health effects;

Int_ID: interventions ID number;

ScatP=Scatterplot of probabilistic simulations of incremental health benefits and incremental costs;

USA: Univariate sensitivity analysis;

CI: 95% Confidence intervals;

SE: Standard errors;

ICERhist: histogram of incremental cost effectiveness ratio;

PSA: Raw simulations from Probability sensitivity analysis;

CEAC: Cost effectiveness acceptability curve

1- Typology of sensitivity analyses undertaken

Sensitivity analysis undertaken	Medium used to report uncertainty data	Numbers of interventions documented by
Univariate	Table of incremental costs and benefits under single parameter changes	5
	Tornado plot of ICERs under single parameter changes	2
Total univariate sensitivity analyses		7
Probabilistic	Raw simulations	2
	Scatterplot of incremental costs versus incremental benefits	1
	Histogram of ICER	1
	Cumulative distribution of ICER (=CEAC)	1
	Confidence intervals around mean incremental costs and benefits	8
	Standard errors around mean incremental costs and benefits	1
Total probabilistic analyses		14
Total interventions with a sensitivity analysis undertaken around cost-effectiveness results		21

Table A1 - Typology of sensitivity analyses undertaken, and medium used to report results in the subset of 21 interventions for which a sensitivity analysis around cost-effectiveness results had been undertaken

2- Value of research when considering all 67 interventions

Method: By using the average of the magnitude of correlation and variances around IHB and IC derived for the subset of 21 interventions with sensitivity analyses output available as underpinning statistical parameters for generating bivariate normal probability distributions for incremental costs and incremental health benefits for those 46 interventions without any sensitivity analysis output.

Using this approach, bivariate normal probability distributions for incremental costs and incremental health benefits were generated for each of the 46 interventions without sensitivity analysis output assuming:

- a standard error for IHB equal to 44% of intervention-specific mean point-estimate for IHB

- a standard error for IC equal to 29% of intervention-specific mean point-estimate for IC

- a correlation coefficient between IHB and IC equal to -0.168

These values, and the estimates underpinning their computations, are provided in the output result file generated by the tool, see user-guide downloadable here).

Variance estimates around IHB and IC (that underpin the generation of uncertainty ranges around pNHE and of estimates of the value of research) derived using this approach are denoted ‘Scenario-based variance estimates’ as opposed to ‘Variance estimates generated from sensitivity analysis results’.

Further information about the method and where these variance estimates can be found in the Output results file is available in the tool’s user-guide, downloadable here:

BMJ Publishing Group Limited (BMJ) disclaims all liability and responsibility arising from any reliance placed on this supplemental material which has been supplied by the author(s)

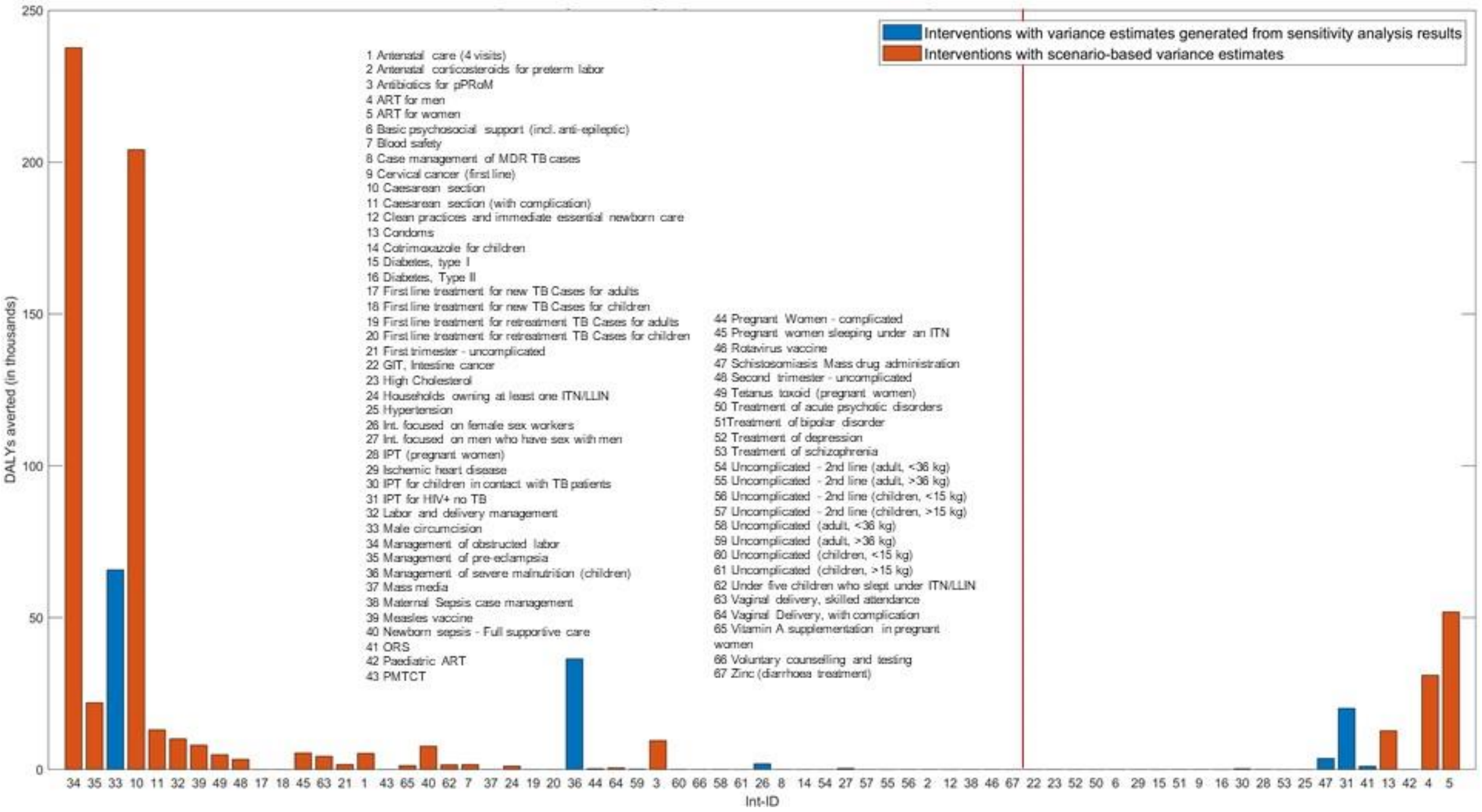

Figure A1: Potential population net health effects (pNHE) from research for all 67 interventions – ranked by expected pNHE

3- Sensitivity Analyses:

Objective: Evaluating whether our top three interventions associated with the largest value of research is sensitive to changes in structural assumptions.

3a- Two main structural assumptions (see Figure 2 in main manuscript)

SA1- If the correlation coefficient between IHB and IC was not specified or could not be extracted from sensitivity analysis output, its value used to simulate IHB and IC as bivariate normal variables was informed by the mean of the correlation coefficients that could be extracted from interventions where uncertainty data came from either univariate sensitivity analysis (USA), PSA simulations or scatterplots (ScatP). In our subset of 21 interventions, the mean of the correlation coefficients extractable from sensitivity analysis output was: -0.168 (this value is provided in the output result file generated by the tool, see user-guide downloadable here).

SA2- For interventions for which uncertainty around IHB and IC was informed by a histogram of ICER or by a CEAC, that is interventions for which only information on the ratio of IC and IHB was available, minimum and maximum values for IHB were set equal to +/- 100% mean.

3b- Results sensitivity to structural assumption SA1

				Base case corr= -0.168		corr=-0.5		corr=+0.5		corr=0	
**Int_ID**	**Sensitivity analysis type and medium**	**Sensitive to SA1**	**Intervention name**	**ranking**	pNHE	ranking	pNHE	ranking	pNHE	ranking	pNHE
33	ScatP	No	Male circumcision	1	65,762	1	65,762	1	65,762	1	65,762
36	USA	No	Community-mgt acute malnutrition (CMAM)	2	36,438	2	36,438	2	36,438	2	36,438
**31**	**CI**	**Yes**	Isoniazid Prev. Therapy for HIV+ no TB	**3**	**20,132**	**3**	**26,049**	**3**	**6,939**	**3**	**16,941**
**47**	**CEAC**	**Yes**	Schistosomiasis Mass drug administration	**4**	**3,667**	**5**	**1,946**	**4**	**6,792**	**4**	**4,554**
**26**	**CI**	**Yes**	Interventions focused on female sex workers	**5**	**1,883**	**4**	**2,454**	**6**	**676**	**5**	**1,590**
41	PSA simulations	No	Oral Rehydration Salts	6	1,072	6	1,072	5	1,072	6	1,072
**27**	**CI**	**Yes**	Interventions focused on men who have sex with men	**7**	**353**	**7**	**459**	**7**	**127**	**7**	**298**
**30**	**CI**	**Yes**	Isoniazid Prev. Therapy for children in contact with TB patients	**8**	**255**	**8**	**329**	**8**	**88**	**8**	**214**
**14**	**SE**	**Yes**	Cotrimoxazole for children	**9**	**12**	**10**	**14**	**10**	**9**	**9**	**11**
**25**	**ICER**	**Yes**	Hypertension mgt	**10**	**5**	**11**	**0**	**9**	**59**	**10**	**15**

**37**	**CI**	**Yes**	Mass media	**11**	**0**	**12**	**0**	**11**	**0**	**11**	**0**
**42**	**CI**	**Yes**	Pediatric ART	**12**	**0**	**9**	**43**	**12**	**0**	**12**	**0**
**43**	**CI**	**Yes**	Prevention Mother to Child transmission of HIV (PMCT)	**13**	**0**	**13**	**0**	**13**	**0**	**13**	**0**
**66**	**CI**	**Yes**	Voluntary counseling and testing	**14**	**0**	**14**	**0**	**14**	**0**	**14**	**0**
8	USA	No	Case management of MDR TB cases	15	0	15	0	15	0	15	0
17	USA	No	First line treatment for new TB Cases for adults	16	0	16	0	16	0	16	0
18	USA	No	First line treatment for new TB Cases for children	17	0	17	0	17	0	17	0
19	USA	No	First line treatment for retreatment TB Cases for adults	18	0	18	0	18	0	18	0
20	USA	No	First line treatment for retreatment TB Cases for children	19	0	19	0	19	0	19	0
46	USA	No	Rotavirus vaccine	20	0	20	0	20	0	20	0
67	PSA simulations	No	Zinc (diarrhea treatment)	21	0	21	0	21	0	21	0

* Mean value of the correlation coefficients extracted from interventions where uncertainty data came from USA, PSA simulations or ScatP (n=10)

Table A2: Changes in ranking of interventions by the value of research when using base case versus alternative values for the correlation coefficient (SA1)

3c- Results sensitivity to structural assumption SA2

				**base case** min-max IHB = [0; mean*2]		min-max IHB = [**0.5*mean**; 1.5*mean]		min-max IHB = [**-0.5*mean**; 2.5*mean]	
**Int_ID**	**Sensitivity analysis type and medium**	**Sensitive to SA2**	**Intervention name**	**ranking**	pNHE	rankin g	pNHE	ranking	pNHE
33	ScatP	No	Male circumcision	1	65,762	1	65,762	1	65,762
36	USA	No	Community-mgt acute malnutrition (CMAM)	2	36,438	2	36,438	2	36,438
31	CI	No	Isoniazid Prev. Therapy for HIV+ no TB	3	20,132	3	20,132	3	20,132
**47**	**CEAC**	**Yes**	**Schistosomiasis Mass drug administration**	**4**	**3,667**	**4**	**5314**	**5**	**1,659**
26	CI	No	Interventions focused on female sex workers	5	1,883	5	1,883	4	1,883
41	PSA simulations	No	Oral Rehydration Salts	6	1,072	6	1,072	6	1,072
27	CI	No	Interventions focused on men who have sex with men	7	353	7	353	7	353
30	CI	No	Isoniazid Prev. Therapy for children in contact with TB patients	8	255	8	255	8	255
14	SE	No	Cotrimoxazole for children	9	12	10	12	9	12

**25**	**ICER**	**Yes**	**Hypertension mgt**	**10**	**5**	**9**	**49**	**10**	**0**
37	CI	No	Mass media	11	0	11	0	11	0
42	CI	No	Pediatric ART	12	0	12	0	12	0
43	CI	No	Prevention Mother to Child transmission of HIV (PMCT)	13	0	13	0	13	0
66	CI	No	Voluntary counseling and testing	14	0	14	0	14	0
8	USA	No	Case management of MDR TB cases	15	0	15	0	15	0
17	USA	No	First line treatment for new TB Cases for adults	16	0	16	0	16	0
18	USA	No	First line treatment for new TB Cases for children	17	0	17	0	17	0
19	USA	No	First line treatment for retreatment TB Cases for adults	18	0	18	0	18	0
20	USA	No	First line treatment for retreatment TB Cases for children	19	0	19	0	19	0
46	USA	No	Rotavirus vaccine	20	0	20	0	20	0
67	PSA simulations	No	Zinc (diarrhea treatment)	21	0	21	0	21	0

Table A3: Changes in ranking of interventions by the value of research when using base case versus alternative values for setting a minimum and maximum value for IHB when only sensitivity analysis data on the ratio of IC and IHB was available (SA2)
